# Ag-Decorated Localized Surface Plasmon-Enhanced Ultraviolet Electroluminescence from ZnO Quantum Dot-Based/GaN Heterojunction Diodes by Optimizing MgO Interlayer Thickness

**DOI:** 10.1186/s11671-016-1701-5

**Published:** 2016-10-29

**Authors:** Cheng Chen, Jingwen Chen, Jun Zhang, Shuai Wang, Wei Zhang, Renli Liang, Jiangnan Dai, Changqing Chen

**Affiliations:** Wuhan National Laboratory for Optoelectronics, Huazhong University of Science and Technology, 1037 Luoyu Road, Wuhan, 430074 Hubei People’s Republic of China

**Keywords:** ZnO, Quantum dots, Heterostructure, Localized surface plasmon

## Abstract

We demonstrate the fabrication and characterization of localized surface plasmon (LSP)-enhanced n-ZnO quantum dot (QD)/MgO/p-GaN heterojunction light-emitting diodes (LEDs) by embedding Ag nanoparticles (Ag-NPs) into the ZnO/MgO interface. The maximum enhancement ration of the Ag-NP-decorated LEDs in electroluminescence (EL) is 4.3-fold by optimizing MgO electron-blocking layer thickness. The EL origination was investigated qualitatively in terms of photoluminescence (PL) results. Through analysis of the energy band structure of device and carrier transport mechanisms, it suggests that the EL enhancement is attributed to the increased rate of spontaneous emission and improved internal quantum efficiency induced by exciton-LSP coupling.

## Background

Semiconductor nanoparticles termed quantum dots (QDs) have drawn wide attention in recent years as light-emitting source for light-emitting diode (LED) applications, whose emission spectrum with narrow linewidth can be tuned by changing the energy bandgaps with the variation in QD sizes and shapes [1–7]. Numerous experiments present in literature on the high-performance electroluminescence (EL) properties of LEDs based on CdS [8, 9], CdSe [10, 11], and PbS [12, 13] colloidal QD thin film. However, the widespread employment of heavy metal ions, particularly Cd and Pb, are a serious hazard to human health as well as to the environment [14]. Therefore, it necessitates alternative approaches for developing QD-LEDs with the heavy-metal free composition. Nontoxic ZnO QDs with a tunable direct wide bandgap and a large exciton binding energy of 60 meV at room temperature are very promising for solid-state LED applications [15, 16]. However, the main issue, the lack of high-quality and stable p-type doing of ZnO due to the strong self-compensation effect of native point defects such as zinc interstitial or oxygen vacancy, still remained. Thus, n-ZnO/p-GaN heterojunction LEDs are offered as an alternative approach due to the low lattice mismatch (1.9 %), similar bandgap energy between ZnO and GaN, and the same wurtzite crystal structure. Nevertheless, the spontaneous polarization of GaN [17–19] and the interfacial energy barrier between ZnO QDs and GaN will necessarily reduce the performance of the device, thus resulting the low EL efficiency of the device. To improve the EL efficiency of the LEDs, localized surface plasmon (LSP) has been introduced into n-ZnO/p-GaN LEDs to improve EL performance of the device. Recently, different metals like Au, Ag, and Pt have been observed to enhance the EL performance due to the enhancement of the internal quantum efficiency [20–26]. More recently, Lu et al. demonstrated a more than 30-fold EL enhancement of Al nanoparticle-decorated n-ZnO nanorod/p-GaN LEDs compared with that of the bare one [27]. Liu et al. showed the devices of LSP-enhanced ZnO/SiO_2_ core/shell nanorod array/p-GaN heterostructure LEDs containing decorated with Ag nanoparticles. In comparison with the bare UV LEDs, the maximum enhancement ration of the Ag nanoparticle-decorated LEDs in EL is sevenfold [28]. However, many reports are mainly focused on the LSP-enhanced LEDs based on ZnO nanorods; there has been no literature concerning the LSP-enhanced EL emission in ZnO QD-based LED structure.

In this paper, we demonstrated the enhanced LSP-induced EL emission intensity in a n-ZnO QD/MgO/p-GaN LEDs. The MgO insulating layer is introduced primarily to modify the energy level alignment in the p-n heterojunction to confine the recombination. Meanwhile, there is a relatively small lattice mismatch of 6.5 % between GaN and MgO [29]. The maximum enhancement ration of the Ag nanoparticle-decorated LEDs in EL is 4.3-fold by optimizing MgO electron-blocking layer (EBL) thickness. To the best of our knowledge, this is the first work to observe the enhanced LSP-induced EL from ZnO QD-based p-i-n heterojunction LEDs to date. The EL origination and corresponding carrier transport mechanisms are investigated qualitatively in terms of photoluminescence (PL) results and energy band diagram in this study.

## Methods

Commercially available p-type Mg-doped GaN epilayer with a c-plane sapphire base was used as the substrate. The carrier concentration of the p-GaN is approximately 4.53 × 10^17^ cm^−3^. The MgO insulating layer directly gown on the pre-cleaned p-GaN/Al_2_O_3_ substrates by pulsed laser deposition (PLD) of an MgO ceramic target (99.99 % purity). Afterwards, colloidal Ag-NPs were spin-coated onto the MgO film. The colloidal Ag-NPs were synthesized in the presence of polyvinylpyrrolidone via a hydrothermal route [30]. ZnO QDs synthesized through a chemical-precipitation approach in the presence of Zn(CH_3_COO)_2_·2H_2_O (99.0 % purity) and NaOH (96.0 % purity), and the reaction process proceeds in detail can be found in previous reports [31, 32]. The as-prepared ZnO QDs (20 mg/mL in ethanol) were uniformly spin-coated on the structure for 30 s at 3000 rpm to yield a complete and uniform ZnO QD thin film and then baked at 80 °C for 20 min to remove the solvent completely. At last, for the ohmic contact for n-ZnO and p-GaN layers, metal In monolayer and bilayer Ni/Au electrodes were made by electron beam evaporation. It is worth noting that because excitons were only located within the near field of the Ag-NPs’ surface leading to resonant coupling. Therefore, a proper MgO thickness is of much importance for optimizing LED performance. The penetration depth (*Z*) of the Ag surface plasmon evanescent field into the MgO dielectric layer is calculated from the following equation [33]: $Z=\lambda /2\pi {\left[\left({\varepsilon}_d^{\prime }-{\varepsilon}_{\mathrm{metal}}^{\prime}\right)/{\upvarepsilon^{\prime}}_d^2\right]}^{1/2}$, where ${\varepsilon}_d^{\prime }$ and ${\varepsilon}_{\mathrm{metal}}^{\prime }$ represent the real parts of the dielectric constants of MgO and Ag, respectively, and *Z* can be calculated as *Z* = 35 nm at an emission wavelength of 360 nm. Therefore, the MgO thickness is controlled within 40 nm, the maximum luminescence enhancement can be obtained by varying MgO thickness, and this will be discussed in detail.

The morphologies and structures of the LSP-enhanced ZnO QD/MgO/p-GaN heterojunction were investigated by field emission scanning electron microscope (FESEM; FEI Nova NanoSEM). The as-prepared ZnO QDs were characterized by high-resolution transmission electron microscopy (HRTEM; FEI Tecnai G20). The current–voltage (I–V) characteristic curves of the devices were acquired using a Keithley 2420 sourcemeter. The PL measurements were recorded under a 325-nm He-Cd laser, and the emission was collected via a HORIBA Jobin-Yvon monochromator. EL spectra were recorded using homemade on-wafer testing and analyzing equipment including a prober system and a spectrograph.

## Results and Discussion

**Fig. 1:**
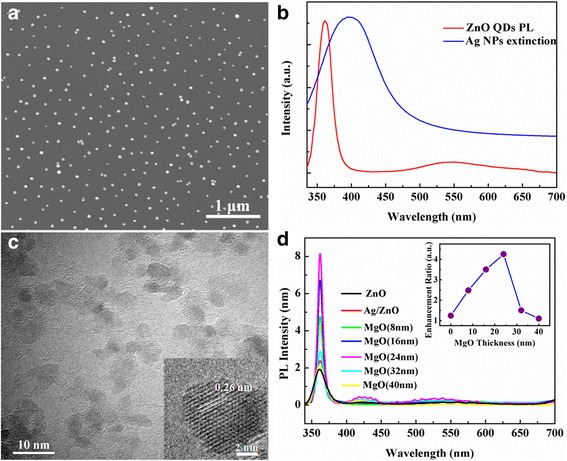
**a** SEM image of the Ag-NPs spin-coated onto the MgO/p-GaN/sapphire film. **b** The extinction spectrum of Ag-NPs and the PL spectrum of ZnO QD film. **c** TEM image of the as-prepared ZnO QDs. The *inset* shows the typical HRTEM image. **d** PL spectra of bare ZnO film, Ag/ZnO film, and MgO/Ag-NP/ZnO composite films with various MgO interlayer thicknesses. The *inset* shows the variation of UV enhancement ration with the MgO interlayer thickness

As shown in Fig. 1a, Ag-NPs are evenly distributed throughout the MgO/p-GaN/sapphire film with an average particle size of ~40 nm and their shapes are nearly spherical. Figure 1b shows the extinction spectra of Ag-NPs, it can be seen that the resonance position of Ag-NP LSP is located at 400 nm, and there is considerable overlap between the broad Ag LSP resonance extinction band and the ZnO UV luminescence, indicating the probability of resonant coupling between Ag LSPs and ZnO excitions. The as-prepared ZnO QDs exhibit ball-like shape, and the mean particle size is 7 nm, as shown in the TEM image of Fig. 1c. It can be seen that the interplanar spacing in the crystalline petal is 0.26 nm in the inset of Fig. 1c, which correspond in the (002) planes of wurtzite ZnO, indicating the good crystallinity of the ZnO QDs. The PL spectra of the MgO/Ag-NP/ZnO composite films with various MgO thickness are shown in Fig. 1d. As the MgO thickness increased, the ZnO UV emission only gradually improved and no obvious variations are found in the visible emission. This phenomenon may be because the introduction of MgO interlay can suppress nonradiative Förster resonant energy transfer processes and thus leads to PL enhancement. Meanwhile, owing to the photon energy of ZnO UV emission is nearly consistent with the Ag-NPs’ LSP resonance (LSPR) energy, so only the UV emission is efficiently enhanced. The PL integrated intensity reached maximum when the thickness of MgO layer is 24 nm, about 4.2-fold in comparison with that the device without Ag-NPs. However, with further increasing in MgO thickness, PL enhancement decreases sharply because of the evanescent wave nature of LSP. The corresponding variation of the enhancement ration of ZnO UV emission with the MgO thickness is illustrated in the inset of Fig. 1d. Thus, the MgO thickness of ~24 nm was selected as the insulating layer for the following LED device.

**Fig. 2:**
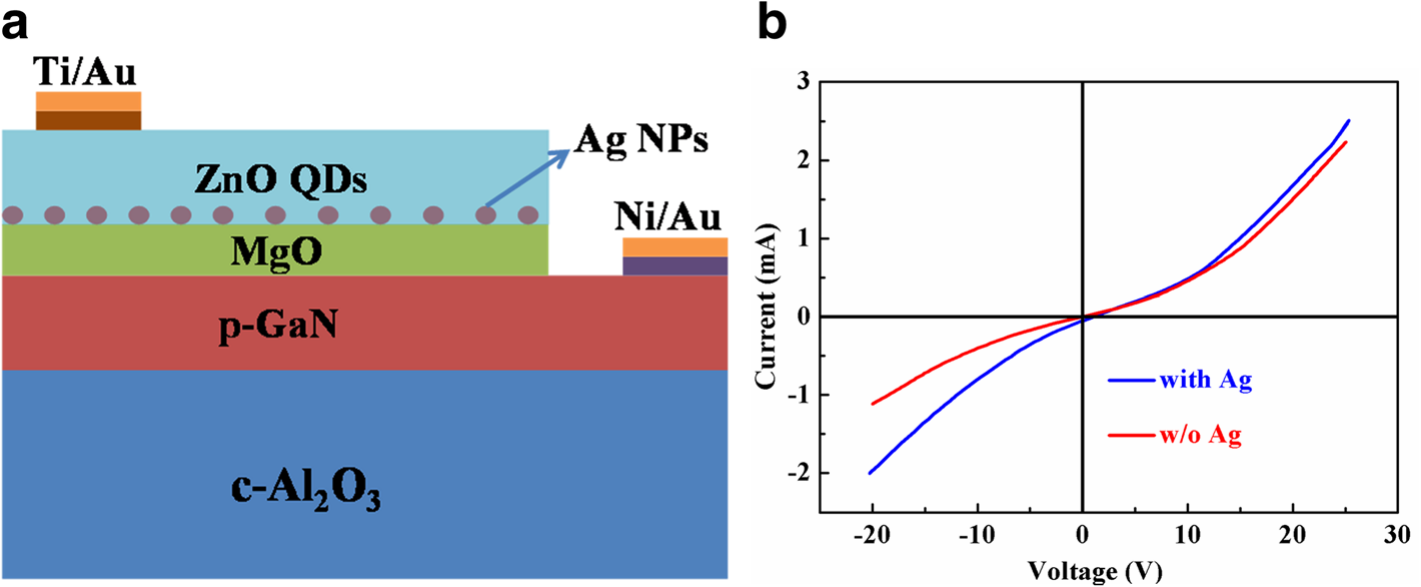
**a** Schematic diagram of the LSP-enhanced ZnO QD/MgO/p-GaN LED device. **b** The I–V characteristic curve of the devices with and without Ag-NPs, and the *inset* shows the characterization of ohmic contacts on each part

Figure 2a shows the schematic device structure of the LSP-enhanced ZnO QD/MgO/p-GaN LEDs. Figure 2b presents nonlinear I–V characteristic curve of the devices with and without Ag-NPs; it can be seen that both exhibit an obvious diode-like rectifying behavior with nearly the same turn-on voltage of about 8.0 V. The inset of Fig 2 shows the perfect I–V linear dependence exhibits good ohmic contact characteristic between a pair of Ni/Au or In electrodes. Compared with the LED without Ag-NPs, the LED with embedded Ag-NPs has relatively lower forward and reverse series resistance. The obvious decrease of the series resistance may result from the creation of more conductive paths owing to the introduction of Ag-NPs, thus resulting in a larger leakage current [34].

**Fig. 3:**
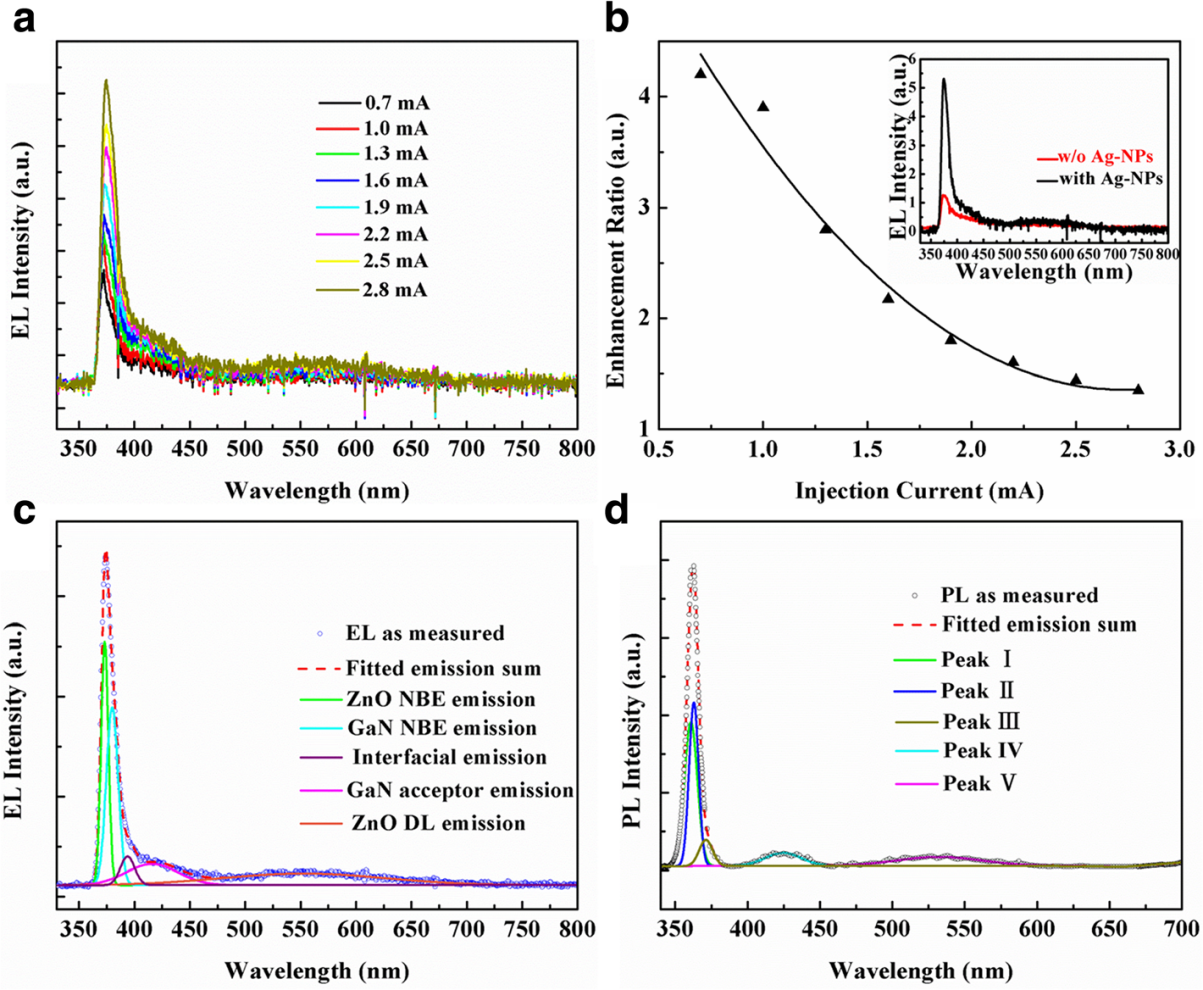
**a** EL spectra of the LSP-enhanced ZnO QD/MgO/p-GaN LED under different forward injection currents. **b** The injection current dependence of the EL enhancement ration for the LED. The *inset* shows EL spectra of the n-ZnO QD/MgO/p-GaN heterojunction LEDs with and without Ag-NPs under an injection current of 0.7 mA. **c** Peak deconvolution of the EL spectra with Gaussian functions obtained at the injection current of 0.7 mA. **d** Gaussian deconvolution analysis of PL spectrum of the ZnO QD/MgO/p-GaN heterojunction with the MgO interlay thickness of 24 nm

Figure 3a illustrates the EL spectra of the LSP-enhanced ZnO QD/MgO/p-GaN heterojunction LEDs with Ag-NPs under different forward injection currents covering from 0.7 to 2.8 mA. It is shown that the EL spectrum is composed of an evident UV emission located at 370 nm and a broad visible emission covering the range from 480 to 650 nm. With increasing of forward injection currents from 0.7 to 2.8 mA, the UV emission increases significantly and grows rapidly to be dominant, while the visible emission has been almost totally suppressed, which is similar to the previous literature [35]. However, the EL enhancement ration of the device as a function of the injection current exhibits an opposite tendency in Fig. 3b. A 4.3-fold EL enhancement is obtained under an injection current of 0.7 mA, and then, EL enhancement ration decreases rapidly with the increase of the injection current. This situation is a result of the screening effect of excess carriers, leading to a weakening of LSP-exciton coupling. The EL spectra of the ZnO QD/MgO/GaN LEDs with and without Ag-NPs were recorded at the same injection current of 0.7 mA (inset of Fig. 3b). Obviously, after insertion of Ag-NPs, only the UV emission is largely enhanced, namely, the radiative recombination is mainly confined in i-ZnO region by constructing p-i-n heterojunction. To further deeply understand the EL emission process, peak deconvolution analysis with Gaussian functions is illustrated in Fig. 3c. It is shown that peak deconvolution of the EL spectrum is composed of five individual peaks, located at 360, 370, 398, 420, and 550 nm. To verify the origination of each EL emission peak, the PL spectrum of the MgO/Ag-NP/ZnO composite films with MgO thickness of 24 nm is investigated by the peak deconvolution with Gaussian functions, which is in accord with the EL peak deconvolution data. Comparing the EL bands to the PL results in Fig. 3d, the five individual peaks mentioned above are successively ascribed to the near band emission (NBE) from the ZnO layer, that of the NBE emission from the GaN, that of interfacial radiative recombination, that of Mg acceptor-related emission of p-GaN, and that of the defect level (DL) emission from the ZnO [32].

**Fig. 4:**
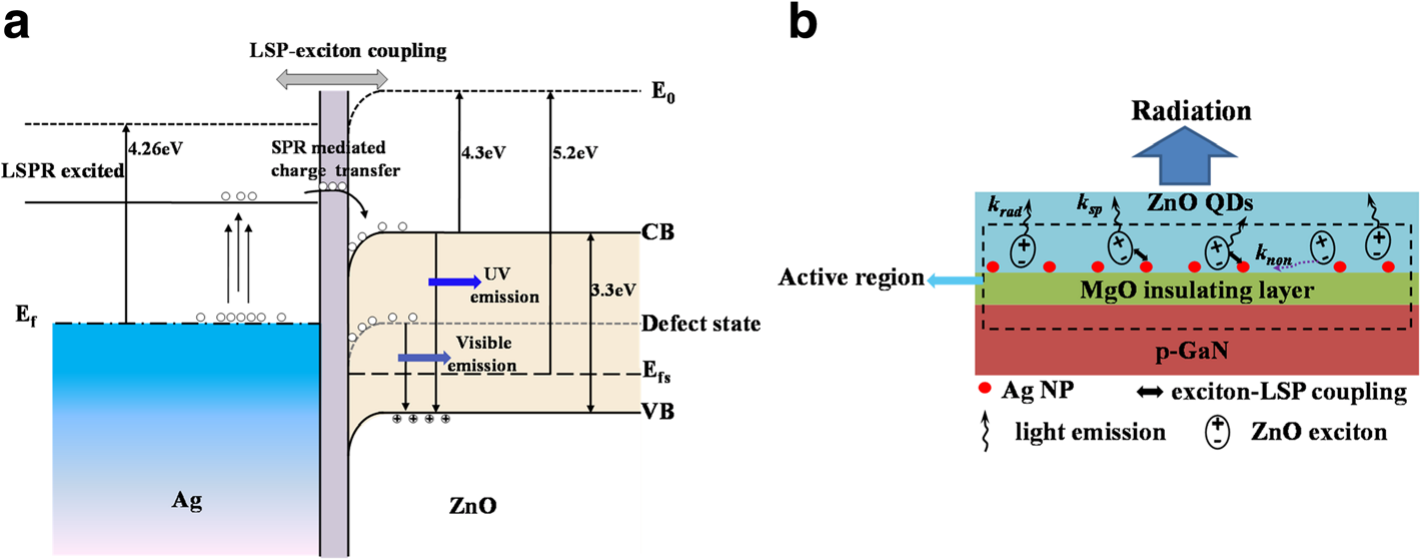
**a** Energy band diagram of the LED showing LSP-exciton coupling. **b** The LSP-enhanced EL process schematic diagram. The *k*_rad_ and *k*_non_ denote the radiative and nonradiative recombination processes, respectively

In order to investigate the reason for the significant enhancement of UV emission, it is essential to understand the energy band structure of the LED and the carriers transfer process between the interfaces. Since the work function of ZnO is about 5.2 eV vs. NHE and its first electron affinity is about 4.3 eV vs. NHE, the work function of Ag is about 4.26 eV vs. NHE, as illustrated in Fig. 4a, this enables the electron transfer from the Ag to ZnO until the two systems achieve the uniform Fermi level, thus resulting in forming a strong polarization-induced local electromagnetic field due to the charge separation [36]. The oscillating electrons in the Ag-NPs can be excited to a high energy level by LSPR. The electrons with higher energy can subsequently transfer from the Ag-NPs to the conduction band of ZnO, causing an increased electron density in the conduction band of ZnO and then an enhanced ZnO NBE emission comparing to the device without Ag-NPs. In this course, high-rate recombination channel occurs between LSP and excitons, as shown in Fig. 4b. Meanwhile, the emerging exciton-LSP coupling rate *k*_sp_ is regarded as a faster recombination rate than the *k*_rad_ and *k*_non_ in ZnO [37]. Therefore, the rate of the spontaneous radiation is increased, leading to the improvement of internal quantum efficiency. It suggests that the EL enhancement is attributed to the increased rate of spontaneous emission and improved internal quantum efficiency induced by exciton-LSP coupling.

## Conclusions

In conclusion, we have demonstrated the fabrication and characterization of LSP-enhanced n-ZnO QD/MgO/p-GaN heterojunction LEDs by embedding Ag-NPs into the ZnO/MgO interface. The maximum enhancement ration of the Ag-NP-decorated LEDs in EL is 4.3-fold by optimizing MgO electron blocking layer thickness. The EL origination was investigated qualitatively in terms of PL results. Through analysis of the band structure of device and carrier transport mechanisms, it suggested that the ZnO EL enhancement was attributed to the increased rate of spontaneous emission and improved internal quantum efficiency induced by exciton-LSP coupling. Due to the significant enhancement of UV emission of the device, it may open a door for pure high-performance ZnO QD-based UV LEDs.

## Abbreviations

Ag-NPs: Ag nanoparticles
EBL: Electron-blocking layer
EL: Electroluminescence
LEDs: Light-emitting diodes
LSP: Localized surface plasmon
NBE: Near band emission
PL: Photoluminescence
QDs: Quantum dots
